# Positionality of Community Health Workers on Health Intervention Research Teams: A Scoping Review

**DOI:** 10.3389/fpubh.2020.00208

**Published:** 2020-06-16

**Authors:** Kiera Coulter, Maia Ingram, Deborah Jean McClelland, Abby Lohr

**Affiliations:** ^1^Mel and Enid Zuckerman College of Public Health, University of Arizona, Tucson, AZ, United States; ^2^University of Arizona Health Sciences Library, University of Arizona, Tucson, AZ, United States

**Keywords:** community health workers, intervention research, participatory research, health intervention, academic-community partnerships

## Abstract

Community health workers (CHWs) are increasingly involved as members of health intervention research teams. Given that CHWs are engaged in a variety of research roles, there is a need for better understanding of the ways in which CHWs are incorporated in research and the potential benefits. This scoping review synthesizes evidence regarding the kinds of health research studies involving CHWs, CHWs' roles in implementing health intervention research, their positionality on research teams, and how their involvement benefits health intervention research. The scoping review includes peer-reviewed health intervention articles published between 2008–2018 in the U.S. A search of PubMed, Embase and CINAHL identified a total of 3,129 titles and abstracts, 266 of which met the inclusion criteria and underwent full text review. A total of 130 articles were identified for a primary analysis of the research and the level of CHWs involvement, and of these 23 articles were included in a secondary analysis in which CHWs participated in 5 or more intervention research phases. The scoping review found that CHWs are involved across the spectrum of research, including developing research questions, intervention design, participant recruitment, intervention implementation, data collection, data analysis, and results dissemination. CHW positionality as research partners varied greatly across studies, and they are not uniformly integrated within all stages of research. The majority of these studies employed a community based participatory research (CBPR) approach, and CBPR studies included CHWs as research partners in more phases of research relative to non-CBPR studies. This scoping review documents specific benefits from the inclusion of CHWs as partners in health intervention research and identifies strategies to engage CHWs as research partners and to ensure that CHW contributions to research are well-documented.

## Introduction

Using the community health worker (CHW) workforce in health promotion programs to reach vulnerable and marginalized populations has become a best practice in addressing health disparities. A CHW is “a frontline public health worker who is a trusted member of and/or has an unusually close understanding of the community served” (1). CHWs work under a variety of job titles, including *promotores de salud*, community health advisors, community health representatives, lay health advisors, and outreach workers. CHWs leverage their deep connections within the community to be a liaison between health services and community residents. In this effort, CHWs assume diverse and wide-ranging responsibilities, including patient outreach, health education and assessments, care coordination, cultural mediation between individuals and social service systems, and individual and community advocacy (2). Studies have shown that CHWs are highly effective in increasing healthcare utilization (3–6), preventive screening (7) and health behavior change (8).

While research on CHW interventions has demonstrated effectiveness in improving health outcomes, CHWs themselves are increasingly incorporated as members of intervention research teams (9, 10). This activity is reflected in the Progress Report of the Community Health Worker (CHW) Core Consensus (C3) Project, a recent national study and consensus-building process to revisit CHW core competencies which added participation in evaluation and research as a new CHW core role and competency (2). The CHW profession encompasses competencies that have potential benefit to health intervention research (2, 10–12). They have a deep understanding of the challenges faced by their communities and can ensure that health interventions address communities' needs. As trusted individuals, CHWs may be well-positioned to involve community members in research studies, particularly among underserved populations (12–14). CHWs can utilize their cultural insights to ensure that intervention implementation and data collection methodologies are responsive to community norms, language(s), and beliefs (12). Scholars have also noted that CHWs' input can be essential to interpreting participants' experiences and perspectives, thus elevating community members' voices within research and improving the quality of the data analysis (10, 15, 16).

Community-based participatory research (CBPR) that seeks to engage community members as partners may be more likely to incorporate CHWs to increase representation of community priorities (2, 17, 18). The CBPR approach intentionally delineates each phase of the research as an opportunity for community engagement, and CHWs' immersion within their communities positions them to represent and/or facilitate the engagement of community members in CBPR studies. While CHWs possess critical assets and skills to participate meaningfully across all phases of research, it is unknown the extent to which researchers engage with CHWs as partners, thus accessing their full scope of practice.

This article describes the results of a scoping review designed to synthesize the nature of CHW involvement across the phases of research, with the overall aim of identifying specific ways in which this workforce can enhance the quality of health intervention studies. The general purpose of a scoping review is to map key concepts underpinning a research area, especially one that is complex and/or understudied (19). In conducting the scoping review, we examined the following questions: (1) What types of research studies involve CHWs? (2) How are CHWs involved across the phases of research? (3) What is the nature of CHW positionality on research teams? (4) In what ways does CHW involvement benefit the quality of health intervention research?

## Materials and Methods

### Search Strategy

An initial review of the Joanna Briggs Institute Database of Scoping Reviews and Implementation Reports, the Cochrane Database of Scoping Reviews, and the Campbell Collection confirmed no existing scoping review on the subject area of CHW roles in intervention research. We collaborated with a medical librarian to design a comprehensive search strategy by developing terms for the concept areas “community health workers,” to identify CHWs working under an array of job titles, and “community-based participatory research,” to ensure that we were identifying studies that were most likely to engage the CHW workforce. Because our search term domains have numerous synonyms, we developed a full list of search terms for each. We then conducted a search for English-language articles in the electronic databases PubMed, Embase, and CINAHL on September 28, 2018.

### Inclusion and Exclusion Criteria

We referred to pre-specified inclusion and exclusion criteria for both title and abstract screening and subsequent full-text review. During title and abstract screening, we applied the following criteria:

1. Articles were peer-reviewed health intervention studies published from 2008–2018
2. Articles included study results
3. Articles included “CHW” (or an alternative name included within our search) within the title and/or abstract
4. Article's description of CHWs aligns with the APHA's definition
5. Articles described interventions within the U.S.

We excluded gray literature, study protocol papers, conference abstracts, formative research (i.e., focus groups, needs assessments), literature reviews, trainings, process evaluations (no outcome data), secondary data analyses, and articles presenting only baseline results of health interventions. We focused on interventions from the U.S. only given that the aforementioned C3 report had newly identified participation in research and evaluation as a new competency for CHWs within the U.S. only. We excluded interventions that involved patient navigators and health educators based upon the CHW Standard Occupational Classification, which differentiates these positions from CHWs (20). We also ensured that the article's description of CHWs aligned with the APHA definition of CHWs (1). If it was also not explicit that the CHWs were from the community served, the article was excluded.

### Study Screening

Two authors independently screened all titles and abstracts and reviewed the selected full-text articles using Covidence, a web-based program to manage literature reviews. Disagreement between reviewers was resolved through direct discussion at all stages.

### Data Extraction

Two reviewers, the first and second author, independently extracted relevant data from the included studies into separate but identical Excel spreadsheets. Within the primary analysis, we noted characteristics of the health intervention, including intervention focus, study design (if the study was a randomized-control trial or not), if the study methodology included a CBPR approach, the target population and the CHW job title CHW (i.e., CHW, lay health worker, *promotora*, etc.). Reviewers then assessed CHW involvement (CHWs involved = 1, CHWs not involved = 0) across the following research phases: identifying the research question; intervention design; instrumentation/measurement design; recruitment/participant eligibility; intervention implementation; data collection; data analysis; and dissemination/action. Lastly, the reviewers sought to ascertain the level of expertise of CHWs by evaluating the described training and experience, with a specific focus on CHW core competencies (2). CHW core competencies refer to core roles and skills that constitute CHWs' full scope of practice such as cultural mediation, providing culturally appropriate health education and relationship building (2). Those studies that hired explicitly experienced CHWs, we interpreted as CHWs proficient in the core competencies. The reviewers then compared the information extracted and resolved any discrepancies in intervention characteristics through reexamination of the article. We calculated the total number of research phases in which CHWs were involved for each article, as well as the percentage of studies that included CHWs in each phase. To explore the benefits of CHW involvement, we conducted a secondary analysis of those articles in which CHWs were involved in 5 or more research phases.

## Results

**Figure 1:**
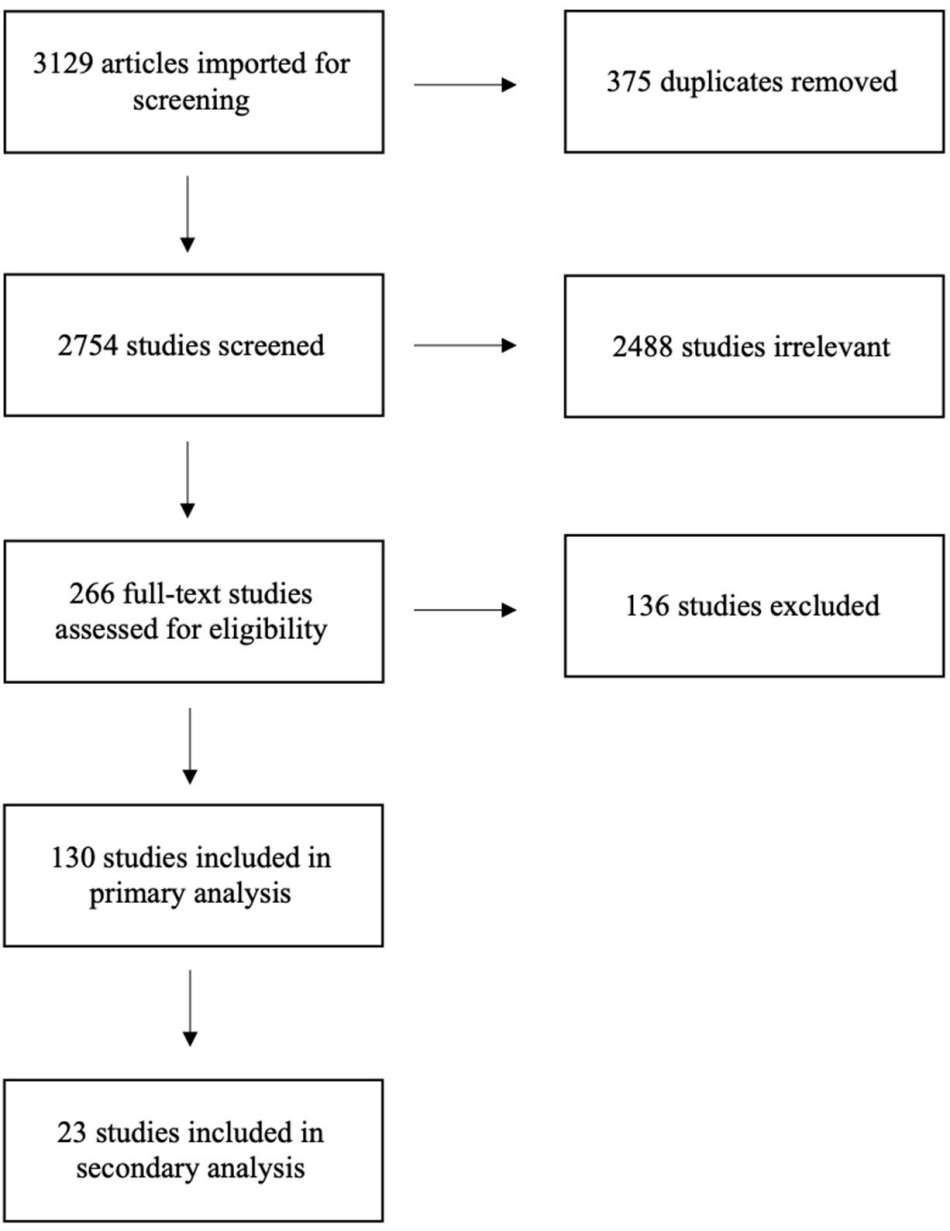
PRISMA Flow Diagram of Scoping Review Process for Examining CHWs in Research.

The initial search resulted in a total of 3,129 articles for review (Figure 1). After removing duplicates in Endnote, we reviewed 2,754 titles and/or abstracts articles against the inclusion criteria to determine eligibility for full-text review. A total of 266 articles underwent full-text review, and 130 articles fitting the criteria were included in the primary analysis (the list of all articles and their full citations is available in Data Sheet 1). For the secondary analysis, we extracted additional information from 23 of the articles which described research projects in which CHWs participated in 5 or more phases.

Table 1 summarizes the characteristics of the 130 health intervention studies, the number of research phases engaging CHWs, and types of CHW training. More than half of the studies (51.5%) focused on disease prevention and promotion and 25.4% of studies targeted individuals with a chronic disease. As expected, the majority (60%) were CBPR studies, while the remaining 40% were non-CBPR studies. Additionally, 43.1% of the studies were randomized-control trials, while the remainder employed quasi-experimental or non-experimental study designs. Across the phases of research, almost all studies (98.5%) utilized CHWs to implement the health intervention. CHWs were also frequently involved in participant eligibility and/or recruitment (57.6%) and data collection (49.2%). CHWs were much less frequently involved in identifying the research question (10.8%), data analysis (2.3%), and research dissemination/action (10.8%).

**Table 1:** Summary of health intervention characteristics, CHW research engagement, and CHW training.^a^

**Characteristic**	***N* = 130**
CBPR, *n* (%)	79 (60.8)
Randomized control trial, *n* (%)	56 (43.1)
**Intervention focus**, ***n*** **(%)**	
Chronic disease	33 (25.4)
Disease prevention and promotion	67 (51.5)
Maternal and child health	10 (7.7)
Substance use	5 (3.8)
Environmental and occupational health	11 (8.5)
Mental health	4 (3.1)
**Phases of research in which CHWs were engaged**, ***n*** **(%)**	
Identifying the research question, *n* (%)	14 (10.8)
Intervention design, *n* (%)	36 (27.7)
Instrumentation and measurement design, *n* (%)	19 (14.6)
Eligibility and/or recruitment, *n* (%)	75 (57.6)
Intervention Implementation, *n* (%)	128 (98.5)
Data collection, *n* (%)	64 (49.2)
Data analysis, *n* (%)	3 (2.3)
Interpretation of results, *n* (%)	16 (12.3)
Dissemination and/or Action, *n* (%)	14 (10.8)
Number of research phases in which CHWs were engaged in all studies, mean (sd)	2.8 (1.9)
Number of research phases in which CHWs were engaged for non-CBPR studies, mean (sd)	2.2 (.94)
Number of research phases in which CHWs were engaged for CBPR studies, mean (sd)	3.3 (2.2)
Number of studies with high CHW involvement engaged in 5 or more phases, *n* (%)	23 (17.7)
**CHW expertise (core compentencies)** ***n*** **(%)**	
Core competency training**	27 (20.8)
Trained but not in core-competencies	44 (33.8)
Experienced in profession	44 (33.8)
No training or not described	15 (11.5)

*CHW, community health worker; sd, standard deviation*.

a *Percentages might not sum to 100 due to rounding or missingness*.

** *Core competencies as defined by the C3 Report*.

In examining CHW expertise, 33.8% of the studies worked with experienced CHWs and an additional 20.8% of the studies trained newly hired CHWs in core competencies. Another 33.8% provided training with CHWs that did not explicitly include core competencies. The remaining studies (11.5%) either didn't describe the training provided or did not mention training or experience of the CHWs. Notably, 64.6% of CBPR studies included CHWs proficient in core competencies, compared to 39.2% among non-CBPR studies. This suggests that participatory researchers may have greater understanding of the relevance of the broader scope of practice in CHW effectiveness. Additionally, the range CHW involvement across phases for non-CBPR studies was one to four, while CBPR studies engaged CHWs in one to nine phases. However, the research goals of the non-CBPR studies were less focused on community engagement and may have been less likely to benefit from CHWs' strengths as a workforce.

Although not documented in Table 1, the target populations of the interventions varied by race and ethnicity, disease focus (i.e., diabetes, hypertension), health behavior (i.e., physical activity), occupation, and/or geographic location. The titles of the CHWs within the interventions were wide-ranging, and included *promotoras*, lay health workers, lay health advisors, community health advisor, community health coaches, community wellness coaches, care guides, resident health advocates, and women's health advocates.

### Secondary Analysis

To identify the characteristics of studies that utilized a broader scope of CHW practice and examine the extent to which the research benefited from CHW involvement, we reviewed those studies in which CHWs engaged in five or more phases (*n* = 23). These studies all described utilizing a CBPR approach. For each study, we documented and synthesized the articles' descriptions of CHW roles and contributions to the quality of the study. Table 2 summarizes the results of the secondary analysis. Notably, in all but four studies, the CHWs were described as experienced or were trained in core competencies as part of the study. Across the studies, CHWs were also trained in intervention delivery, data collection methodologies, research principles, health conditions/diseases, program development, advocacy, and coalitions/networking.

**Table 2:** CHW involvement in phases of research and benefits across high CHW involvement studies.

**Author**	**Intervention focus**	**CHW term**	**Target population**	**Number of research phases with CHW participation**	**CHW experience & training**	**Benefits of CHW involvement**
Arredondo et al. (21)	Disease Prevention & Promotion	promotora	U.S.-Mexico border region community	9	• experienced CHWs • walkability assessments/park audits • advocacy • developing physical activity programs • motivating behavioral change	• CHWs encourage individual/community advocacy for sustained systems/environmental change. • Through their participation and scope of practice, CHWs increased potential for the intervention's translation to practice or sustainability. • Increased capacity of CHWs creates a lasting community resource.
Bush et al. (22)	Environmental Health	promotora	Latino forest workers	8	• core competencies • popular education • communication • hazards of forest work • employer responsibilities • resources • curriculum delivery	• CHWs ensured that research approach considered broader community context. • CHWs were able to access broader social/kin networks for recruitment. • CHWs negotiate the involvement of structurally vulnerable communities in research. • CHWs encourage individual/community advocacy for sustained systems/environmental change. • Increased capacity of CHWs creates a lasting community resource.
Cramer et al. (23)	Maternal & Child Health	CHW	pregnant women	5	• experienced CHWs • prenatal health coaching	• CHW participation ensured community acceptability of the intervention. • Research leverages experienced CHWs embedded within community rather than hiring new CHWs specifically for research.
Furman et al. (24)	Maternal & Child Health	CHW	African American mothers	5	• experienced CHWs • no other training mentioned	• Research leverages experienced CHWs embedded within community rather than hiring new CHWs specifically for research. • Increased capacity of CHWs creates a lasting community resource.
Harvey et al. (25)	Disease Prevention & Promotion	CHW	African American and Latina women	6	• core competencies • hypertension and type 2 diabetes • disease screening processes • diabetes self-management • curriculum delivery • data collection procedures	• CHWs ensured research approach considered broader community context. • Through their participation and scope of practice, CHWs increased potential for the intervention's translation to practice or sustainability.
Ingram et al. (26)	Disease Prevention & Promotion	promotora	Mexican-American moms in border region	8	• experienced CHWs • core competencies training (upon hire)	• CHW participation ensured community acceptability of the intervention. • CHWs validated study findings on the basis of their lived experiences. • CHWs encourage individual/community advocacy for sustained systems/environmental change. • Research leverages experienced CHWs embedded within community/organizations rather than hiring new CHWs specifically for research.
Ingram et al. (27)	Disease Prevention & Promotion	promotora	Latinos in the border region	7	• experienced CHWs • community advocacy	• CHW connection to community resources ensured maximized participant benefit from the intervention. • CHWs encourage individual/community advocacy for sustained systems/environmental change. • Research leverages experienced CHWs embedded within community/organizations rather than hiring new CHWs specifically for research.
Islam et al. (28)	Chronic disease	CHW	Bangladeshi adults with T2DM	6	• CHW training not detailed	• CHWs ensured that research approach considered broader community context. • CHW participation ensured community acceptability of the intervention. • CHW positionality enhances retention and follow-up. • CHW connection to community resources maximized participant benefit from the intervention.
Islam et al. (29)	Disease Prevention & Promotion	CHW	Korean adults at risk for diabetes	6	• core competencies • study protocol • curriculum delivery • mental health • motivational interviewing	• CHW participation ensured community acceptability of the intervention. • CHW positionality enhances retention and follow-up. • CHW connection to community. resources maximized participant benefit from the intervention.
Islam et al. (30)	Disease Prevention & Promotion	CHW	Sikh adults at risk for diabetes	7	• core competencies • study protocol and methods • curriculum delivery • mental health • motivational interviewing • basic action planning	• CHW participation ensured community acceptability of the intervention. • CHW positionality enhances retention and follow-up. • CHW connection to community resources maximized participant benefit from the intervention.
Kutcher et al. (31)	Disease Prevention & Promotion	CHW	Hispanic communities	8	• experienced CHWs • advocacy training • CHWs roles in coalitions • developing community action plans • community assessment strategies	• CHWs brought community perspectives and voices to research process. • CHWs encourage individual/community advocacy for sustained systems/environmental change. • Research leverages experienced CHWs embedded within community/organizations rather than hiring new CHWs specifically for research. • Increased capacity of CHWs creates a lasting community resource.
Marín et al. (32)	Environmental Health	promotora	Latino poultry workers	5	• core competencies • research principles • curriculum delivery • study protocol and methods	• CHW participation ensured community acceptability of the intervention. • CHWs negotiate the involvement of structurally vulnerable communities in research. • CHWs encourage individual/community advocacy for sustained systems/environmental change. • Increased capacity of CHWs creates a lasting community resource.
Marrone et al. (33)	Chronic disease	CHW	Latino adults with hearing loss	6	• experienced CHWs • hearing loss • communicating with individuals/families with hearing loss	• CHW participation ensured community acceptability of the intervention. • Research leverages experienced CHWs embedded within community/organizations rather than hiring new CHWs specifically for research.
Messias et al. (15)	Disease Prevention & Promotion	promotora	Mexican-origin women	5	• core competencies • curriculum delivery • research principles • data collection procedures	• CHW participation ensured community acceptability of the intervention. • CHWs were able to access broader kin/social networks for recruitment. • CHW positionality enhances retention and follow-up. • CHWs brought community perspectives and voices to research process. • CHW connection to community resources ensured maximized participant benefit from the intervention.
Michael et al. (34)	Disease Prevention & Promotion	CHW	Latino and African American adults	7	• core competencies • research principles • health promotion and disease prevention • study protocol and methods	• CHWs brought community perspectives and voices to research process. • CHWs encourage individual/community advocacy for sustained systems/environmental change. • Increased capacity of CHWs creates a lasting community resource.
Minkler et al. (35)	Environmental Health	promotora	residents of Old Town National City	7	• experienced CHWs • advocacy training • land use, air quality, and energy	• CHWs brought community perspectives and voices to research process. • CHWs validated study findings on the basis of their lived experiences. • CHWs encourage individual/community advocacy for sustained systems/environmental change. • Research leverages experienced CHWs embedded within community/organizations rather than hiring new CHWs specifically for research. • Increased capacity of CHWs creates a lasting community resource.
Moore et al. (36)	Substance Use	promotora	Latino day laborers	5	• experienced CHWs • study protocol and methods, • curriculum delivery	• CHW participation ensured community acceptability of the intervention • CHWs were able to access broader kin/social networks for recruitment. • CHWs negotiate the involvement of structurally vulnerable communities in research. • CHW positionality enhances retention and follow-up. • Research leverages experienced CHWs embedded within community/organizations rather than hiring new CHWs specifically for research.
Nicolaidis et al. (37)	Mental health	promotora	Latina IPV survivors	7	• experienced CHWs • mental health • motivational interviewing	• CHW connection to community resources ensured maximized participant benefit from the intervention.
Rios-Ellis et al. (38)	Maternal & Child Health	promotores	Latina mothers	5	• study protocol and methods • maternal-child health educational content	• CHW connection to community resources ensured maximized participant benefit from the intervention. • Increased capacity of CHWs creates a lasting community resource.
Rios-Ellis et al. (39)	Disease Prevention & Promotion	promotores	Latino families	5	• core competencies • participant outreach • curriculum delivery • data collection procedures • basic evaluation methods	• CHW participation ensured community acceptability of the intervention
Schwartz et al. (40)	Disease Prevention & Promotion	promotores	Hispanic families in SW Idaho	5	• training not described	• CHWs were able to access broader social/kin networks for recruitment.
Simonsen et al. (41)	Disease Prevention & Promotion	CHW “community wellness coaches”	women of color	6	• motivational interviewing • gender norms • obesity management • data collection procedures	• CHWs negotiate the involvement of structurally vulnerable communities in research. • Through their participation and scope of practice, CHWs increased potential for the intervention's translation to practice or sustainability • CHWs encourage individual/community advocacy for sustained systems/environmental change. • Increased capacity of CHWs creates a lasting community resource.
Suarez et al. (42)	Substance Use	promotores	Latino smokers	6	• experienced CHWs • data collection procedures • leadership/organization/interpersonal relationships∙ smoking dependence and cessation • cultural competence • curriculum delivery	• CHW participation ensured community acceptability of the intervention. • CHW positionality enhances retention and follow-up. • Increased capacity of CHWs creates a lasting community resource. • Research leverages experienced CHWs embedded within community/organizations rather than hiring new CHWs specifically for research.

*CHW, community health worker; T2DM, type 2 diabetes mellitus; IPV, intimate partner violence; SW, southwest*.

Our examination of the 23 studies identified 12 distinct benefits of CHW involvement throughout the research process. In many of the studies, CHWs were vital to developing study approaches, methodologies, and interventions that were appropriate for the communities served. More specifically, CHWs *increased the research team's awareness of the broader community context* and social determinants of health impacting the daily lives of potential study participants (22, 25, 28). Bush et al. (22) noted the ways in which CHWs increased researchers' awareness to issues facing Latino forest workers (i.e., wage theft, immigration status, land-lord tenant retaliation, etc.) that could influence their capacity to prioritize the occupational safety concerns that were the focus of the intervention. In the study conducted by Messias et al. (15), CHWs provided researchers with crucial information regarding participants' family care-giving responsibilities, employment obligations, and transportation needs which allowed for effective planning and scheduling of intervention sessions. The articles also demonstrated that *CHWs contributed to the community acceptability of interventions*, particularly in ensuring the cultural congruence of the intervention and identifying culturally relevant modes of intervention delivery (15, 23, 26, 28–30, 32, 33, 36, 39, 42). CHWs implementing the *Salud S*í (Health Yes) intervention were responsible for refining intervention strategies to respond to the needs and characteristics of study participants, such as incorporating spirituality to address depression (26). In other studies, CHWs provided their insights and suggestions to adapt already developed curriculums. Moore et al. (36) explained how the CHWs made changes to the intervention manual to better incorporate Latino cultural values, such as familism, and bring awareness to important stressors confronted by day laborers (i.e., acculturative stress, discrimination, and poverty). In Suarez et al. (42), CHWs identified community settings where they could effectively engage Latino smokers and deliver health education (churches, Latino-owned businesses, home visits, Consulate of Mexico, etc.).

The studies underscored CHWs' ability to engage vulnerable or hard-to-reach populations in research, particularly ethnic-minority populations. This was often achieved by CHWs *accessing their broader social and/or kin networks for participant recruitme*nt (15, 22, 36, 40). Recruitment efforts benefitted from the existing trust and rapport CHWs had with their community members. Messias et al. (15) documented how CHWs identified participants from their existing social networks within schools, churches, work, and the broader community. Furthermore, as noted by Bush et al. (22), CHWs tapped into their kinship networks, facilitating contact with large numbers of forest workers. The studies also demonstrated that *CHWs negotiated the inclusion of structurally vulnerable communities in research* (22, 32, 36, 41), referring to populations whose positionality imposes physical or emotional suffering in patterned ways (43). This was particularly evident among interventions targeting low-wage laborers (i.e., forest workers and poultry workers). Sustaining the participation of these populations in the intervention required the efforts and capabilities of the CHWs. More specifically, Bush et al. (22) acknowledged that the CHWs' cultural knowledge, language fluency, and rapport were critical in engaging immigrant forest workers with deeply embedded fears related to immigration status. Similarly, Marín et al. (32) documented that their CHW program provided a safe environment for immigrant poultry workers to learn more about their rights to a safe workplace and advocate for their occupational safety, despite palpable fears of workplace retaliation.

Following recruitment, CHW positionality within the community *enhanced participant retention in the study, ongoing data collection and follow-up* (15, 28–30, 36, 42). CHWs achieved this by developing trust and rapport with participants that enabled them to recognize and negotiate potential barriers to participation, as well as engendered a desire among participants to complete study processes. For example, researchers attributed high levels of participant retention and compliance with accelerometer measurements in a physical activity intervention to CHW continuous communication with participants (15). CHWs' ability to engage and retain participants throughout the study *brought community perspectives and voices to research process* (15, 31, 34, 35). This was apparent in interventions involving CHW-led advocacy efforts. Kutcher et al. (31) described how CHWs were integral to including community residents impacted by health disparities in health coalitions, thereby providing a voice for stakeholders who traditionally lack power. CHWs were able to facilitate coalition meetings where there was equitable participation among community members, local agencies, and officials. CHWs also elevated community voices through data collection efforts. In an environmental justice initiative, CHWs served as “co-researchers” and led a survey of community residents to capture their concerns and priorities (i.e., asthma, land use, affordable housing, etc.), which later shaped local policy changes (35).

The few studies that involved CHWs in the data interpretation process demonstrated that they were able to explain or *validate study findings based on their common experience* with the study population (26, 35). CHWs in Ingram et al.'s (26) study explained that women who initiated physical activity during the intervention were able to do so because the program created a culturally acceptable space for women to congregate. Without the organized classes, this activity was difficult to maintain. In an effort to enact policy changes consistent with the community's needs, Minkler et al. (35) described how CHWs presented to the City Council the results of a survey they implemented with community residents, which they supported by sharing their experiences as community members and mothers.

In several of the studies, CHW involvement *increased the potential for the intervention's translation to practice or sustainability*, and this finding applied to both program and systems level interventions (21, 25, 41). CHWs helped ensure that the intervention utilized appropriate strategies delivered in an appropriate fashion, taking into consideration the larger context of intervention delivery and the participants' lives. The CHW-driven advocacy efforts initiated as part of REACH projects were more likely to be sustained because the CHWs successfully engaged business owners in prioritizing and implementing changes (25). Similarly, the Arredondo et al. (21) study documented an increased intention to use park facilities among community members after CHWs worked with youth to identify, advocate for, and attain structural changes. On the programmatic level, the Detroit Department of Health continued to employ CHWs and outreach strategies after demonstrating the effectiveness designed by the CHWs in the HC project (25). Notably, an intervention's sustainability was *maximized when the research leveraged experienced CHWs* that were already embedded in community or local organizations, rather than hiring new CHWs specifically for research (23, 24, 26, 27, 31, 33, 35, 36, 42). This is largely because CHWs could incorporate intervention activities into their existing work after the conclusion of the study. In a breastfeeding intervention, the CHWs continued to use curricular models within their existing MomsFirst programming (24).

Importantly, CHWs' connections to community resources helped to *maximize participants' benefit from the intervention* (15, 27–30, 37, 38). Participants were connected to a broader range of community resources more efficiently and also frequently received ongoing services from these entities. CHWs linking participants to community resources (i.e., food stamps, English language programs, etc.) were also cited as reasons for high feasibility and/or acceptability of interventions (28–30). CHWs connected participants to needed services even when not a stated objective of the intervention. For example, within a breastfeeding intervention for Latina mothers, participants reported an increased understanding of where to get help for post-partum depression because the CHW shared her knowledge of relevant services (38).

CHW involvement *encouraged individual/community advocacy to enact sustained health-promoting individual and system level changes* (21, 22, 26, 27, 31, 32, 34, 35, 41). The interventions focusing on occupational safety empowered immigrant workers to advocate for better working conditions (22, 32). Despite notable fears and susceptibility to retaliation among the workers, the support and information from the CHWs empowered them to address workplace hazards such as notifying their supervisors (22, 32). In other studies, CHWs mobilized community members, organizations, and policy-makers to implement policy-systems-environment (PSE) strategies. CHWs encouraged community members to think ecologically about their health and identify advocacy-oriented solutions to improve community social determinants of health. CHWs also modeled behaviors for participating in advocacy coalitions so to support the capacity of community members to work with local representatives/officials to enact PSE changes (31). The organization efforts of the CHWs led to a variety of individual and community changes, such as restorations of a local park (21), improvements to neighborhood conditions, enhanced community opportunities, better access to services (27), increased access to healthy foods, and the adoption of policies to integrate physical activity opportunities into schools (31).

Finally, the increased capacity of CHWs, through their training and increased experience, *created a lasting community resource* (21, 22, 24, 31, 32, 34, 35, 38, 41, 42). As described by Bush et al. (22), after the occupational safety intervention, the CHWs' participation in the program promoted their leadership skills and established them as recognized resources within the community. The CHWs' experiences in research empowered them to take on additional roles, responsibilities, or new jobs. After their demonstrated success in advocacy, Kutcher et al. (31) mentioned that the CHWs adopted new roles facilitating coalition meetings, collaborating with local officials, and representing the project in marketing/communications efforts. At the end of the occupational safety program, Marín et al. (32) noted the promoters were provided new opportunities; one was later hired for a local literacy project, and two others were employed by a worker center supporting low-wage immigrant workers.

## Discussion

Thorough review of CHWs' activities within health intervention research provides important insights for community-academic research teams regarding the breadth of roles CHWs can assume within research and how they can strengthen health research initiatives. Overall, the results of the primary and secondary analysis revealed that CHW participation in health intervention research is diverse, in terms of the kinds of studies they are involved (i.e., study design and focus), their roles in the research process and their positionality on the research team. The majority of studies, and particularly those that engaged CHWs in more phases of the research applied a community based participatory research approach. This finding is consistent with the C3 report's and other scholars' assessments that CHWs facilitate community participation and representation in health research (2). However, a substantial percentage of the studies were not participatory, suggesting that both participatory and non-participatory researchers recognize the relevance of CHWs to health interventions. What did differentiate CBPR and non-CBPR studies was the range of research phases with CHW involvement. CHWs were involved in up to 9 phases within CBPR studies, relative to up to 4 within non-CBPR studies. This result indicates that CBPR studies are more likely to integrate CHWs across the entirety of research process, beginning from identification of the health issue to dissemination of the results.

In nearly all the studies, CHWs were responsible for implementing the intervention under study, and those studies that employed CHWs in this role alone are perhaps better able to distinguish the effectiveness of the CHW-facilitated intervention or the CHW workforce in addressing a particular health issue (i.e., glycemic control, hypertension, etc.). It is not surprising that intervention implementation, participant recruitment, and data collection, defined as discrete phases of research in the CBPR-framework, were the research phases with the highest CHW involvement across the studies. These phases of research involve CHWs in direct interaction with study participants to encourage their participation in the study, facilitate their engagement in the study, or talk with them about their health. Given CHWs' connections within their communities and their effectiveness in engaging with community members, it makes sense that CHWs performed these activities most frequently.

Conversely, CHWs were least involved in identifying the research question, data analysis, and research dissemination. This finding represents lost opportunities for ensuring that not only the research focus, but also research findings, are relevant to communities. Researchers would need to engage CHWs as research partners early in the process if they were to be included in defining the research question and ensuring that the interventions address relevant health issues. As underscored in the secondary analysis, CHWs can also aid in data analysis by explaining study findings, interpreting the voices and perspectives of the study participants, and further validating the data based on their lived experiences. Very few of the studies involved CHWs in a dissemination/action phase of the research, which is unfortunate. This phase is intrinsic to CBPR in ensuring that research contributes to social change (44). While it is possible that these research projects continued into an action phase not reported in the article, it is also the most difficult phase of research and the one for which researchers are least prepared. This may be the major argument for including CHWs as full members on research teams so that they are well-positioned to carry the research forward into community action.

This aspect of CHW involvement is related to their positionality on research teams and the power dynamics between community and academic partners that limit or maximize CHWs' contributions to research. The articles provide several strategies for including CHWs and building their capacity in a partnership role. In some cases, researchers invited CHWs to sit on the decision-making body of the research team, such as the community action board (CAB) or steering committee. This inclusion formalized their leadership position among academics and other stakeholders. As recognized leaders and stakeholders, CHWs are ideally positioned to share their knowledge of community during the formative phases of the study to inform the research approach at the outset. The projects also trained CHWs in research methodologies. While these trainings were frequently not described in-depth, a co-learning environment in which the mutual and shared expertise is valued among all partners would certainly facilitate recognition of CHW contributions. Additionally, CHWs that were incorporated across the phases of research had more opportunities to improve research processes and ensure community benefit. Because CHWs instinctively and are trained to prioritize community interests, they are more likely to identify and address ethical issues related to research that might otherwise go unrecognized (18).

The inclusion of CHWs did not go without challenges. Researchers noted some difficulty in CHWs adhering to research protocols due to concerns of maintaining rapport with community members. One academic-community partnership discussed CHWs encountering tension between fidelity to procedures of randomized-control trials and community norms (15). Another research team described differing goals between academic and community partners (including CHWs), where academic partners prioritized data and community partners prioritized funding and policy (24). While it is important that CHWs are trained in research ethics and procedures, the current study's results highlight how CHWs' knowledge of the community is integral to conducting successful research. For example, Furman et al. (24) explained how some of the staff were hesitant to endorse the research project due to conflicts with on-the-ground realities of the community members served. Thus, if CHWs are challenged by the research protocol, that could signal potential incongruence with community practices that the research team should address. Furthermore, it was clear that research teams valued the community rapport CHWs possessed, but some authors described how some CHWs faced difficulties in leveraging connections outside of their social networks (15, 22). Also, a few studies documented CHWs facing personal conflict between their responsibilities as CHWs engaged in research and their obligations to spouses/family (32, 35).

## Limitations

The scoping review is also characterized by certain limitations. Our knowledge of the CHWs' involvement within the included studies is limited to what was documented in the articles. Thus, if CHWs' participation was not thoroughly described or underreported by the author(s), it was not reflected in our results. We did not seek to evaluate the quality of the research and did not compare it to similar research that did not use CHWs. Also, we did not examine individual health outcomes or community outcomes which we hope would be improved with CHW involvement. Our scoping review does not establish the desired benefits, rather it is an attempt to synthesize lessons learned from a broad variety of research studies and approaches. Lastly, these results do not capture lessons to be learned from research interventions with CHWs outside the U.S. Future research should examine the roles of CHWs within health intervention research globally.

## Conclusion

This scoping review highlights the potential benefits of incorporating CHWs as partners in health intervention research studies. Our findings demonstrate that CHWs can improve the quality of research not only in CBPR studies that seek to engage community members in the research process, but also in non-CBPR studies, including those utilizing experimental designs. We found that CHWs inform study design to consider contextual factors, improve the content and delivery of health interventions, and validate and explain research findings and most importantly, both insure and increase the benefits of research for the individuals and communities involved.

## Author Contributions

KC lead the development of the manuscript. KC and MI collaborated in developing the inclusion/exclusion criteria, conducting article screening, data analysis, and drafting of the manuscript. DM directed the development of the search strategy, implemented the search in the databases, and lead the writing of the methods section of the manuscript. AL edited continuous iterations of the manuscript draft and provided input on the direction of the data analysis.

## Conflict of Interest

The authors declare that the research was conducted in the absence of any commercial or financial relationships that could be construed as a potential conflict of interest.

## Supplementary Material

Data Sheet 1 Data extraction table for primary analysis articles to account for some spelling errors, more accurate listings of some of the target populations of the intervention studies, and accuracy in dates of publication.

## References

[1] American Public Health Association (APHA) Community Health Workers (2019). Available online at: https://www.apha.org/apha-communities/member-sections/community-health-workers (November 1, 2019).

[2] Rosenthal EL Rush CH Allen C Progress Report of the Community Health Worker (CHW) Core Consensus (C3) Project: Building National Consensus on CHW Core Roles, Skills, and Qualities. (2016) Available online at: http://files.ctctcdn.com/a907c850501/1c1289f0-88cc-49c3-a238-66def942c147.pdf?ver=1462294723000

[3] Pérez-Escamilla R Damio G Chhabra J Fernandez ML Segura-Pérez S Vega-López S. Impact of a community health workers–led structured program on blood glucose control among Latinos with type 2 diabetes: the DIALBEST trial. Diabetes Care. (2015) 38:197–205 10.2337/dc14-0327 25125508 PMC4302259

[4] Ursua RA Aguilar DE Wyatt LC Trinh-Shevrin C Gamboa L Valdellon P. A community health worker intervention to improve blood pressure among Filipino Americans with hypertension: a randomized controlled trial. Prev Med Rep. (2018) 11:42–8. 10.1016/j.pmedr.2018.05.002 29984137 PMC6030569

[5] Spencer MS Rosland AM Kieffer EC Sinco BR Valerio M Palmisano G. Effectiveness of a community health worker intervention among African American and Latino adults with type 2 diabetes: a randomized controlled trial. Am J Public Health. (2011) 101:2253–60. 10.2105/AJPH.2010.300106 21680932 PMC3222418

[6] Kim K Choi JS Choi E Nieman CL Joo JH Lin FR. Effects of community-based health worker interventions to improve chronic disease management and care among vulnerable populations: a systematic review. Am J Public Health. (2016) 106:e3–28. 10.2105/AJPH.2015.302987a 26890177 PMC4785041

[7] Han HR Song Y Kim M Hedlin HK Kim K Ben Lee H. Breast and cervical cancer screening literacy among Korean American women: a community health worker–led intervention. Am J Public Health. (2017) 107:159–65. 10.2105/AJPH.2016.303522 27854539 PMC5308166

[8] Koniak-Griffin D Brecht ML Takayanagi S Villegas J Melendrez M Balcázar H. A community health worker-led lifestyle behavior intervention for Latina (Hispanic) women: feasibility and outcomes of a randomized controlled trial. Int J Nurs Stud. (2015) 52:75–87. 10.1016/j.ijnurstu.2014.09.005 25307195 PMC4277872

[9] Rosenthal EL Wiggins N Ingram M Mayfield-Johnson S De Zapien JG. Community health workers then and now: an overview of national studies aimed at defining the field. J Ambul Care Manage. (2011) 34:247–59. 10.1097/JAC.0b013e31821c64d7 21673523

[10] Hohl SD Thompson B Krok-Schoen JL Weier RC Martin M Bone L. Characterizing community health workers on research teams: results from the centers for population health and health disparities. Am J Public Health. (2016) 106:664–70. 10.2105/AJPH.2015.302980 26794157 PMC4986058

[11] Brownstein JN Hirsch GR Rosenthal EL Rush CH. Community health workers “101” for primary care providers and other stakeholders in health care systems. J Ambul Care Manage. (2011) 34:210–20. 10.1097/JAC.0b013e31821c645d 21673520

[12] Johnson CM Sharkey JR Dean WR St John JA Castillo M. Promotoras as research partners to engage health disparity communities. J Acad Nutr Diet. (2013) 113:638–42. 10.1016/j.jand.2012.11.014 23375463 PMC3633728

[13] Choi E Heo GJ Song Y Han HR. Community health worker perspectives on recruitment and retention of recent immigrant women in a randomized clinical trial. Fam Community Health. (2016) 39:53–61. 10.1097/FCH.0000000000000089 26605955 PMC4662073

[14] Larkey LK Gonzalez JA Mar LE Glantz N. Latina recruitment for cancer prevention education via community based participatory research strategies. Contemp Clin Trials. (2009) 30:47–54. 10.1016/j.cct.2008.08.003 18775798

[15] Messias DK Parra-Medina D Sharpe PA Treviño L Koskan AM Morales-Campos D. Promotoras de Salud: roles, responsibilities, and contributions in a multi-site community-based randomized controlled trial. Hisp Health Care Int. (2013) 11:62–71. 10.1891/1540-4153.11.2.62 24695944 PMC3970723

[16] Vaughn LM Whetstone C Boards A Busch MD Magnusson M Määttä S. Partnering with insiders: a review of peer models across community-engaged research, education and social care. Health Soc Care Community. (2018) 26:769–86. 10.1111/hsc.12562 29512217

[17] Cupertino AP Suarez N Cox LS Fernández C Jaramillo ML Morgan A. Empowering promotores de salud to engage in community-based participatory research. J Immigr Refug Stud. (2013) 11:24–43. 10.1080/15562948.2013.759034 25705141 PMC4335649

[18] Smith SA Blumenthal DS. Community health workers support community-based participatory research ethics: lessons learned along the research-to-practice-to-community continuum. J Health Care Poor Underserved. (2012) 23(Suppl. 4):77–87. 10.1353/hpu.2012.0156 23124502 PMC3586526

[19] Arksey H O'Malley L Scoping studies: towards a methodological framework. Int J Soc Res Methodol. (2005) 8:19–32. 10.1080/1364557032000119616

[20] Malcarney MB Pittman P Quigley L Seiler N Horton KB Community Health Workers: Health System Integration, Financing Opportunities, and the Evolving Role of the Community Health Worker in a Post-Health Reform Landsacape (2015). Available online at: https://hsrc.himmelfarb.gwu.edu/sphhs_policy_workforce_facpubs/11/ (April 15, 2019).

[21] Arredondo E Mueller K Mejia E Rovira-Oswalder T Richardson D Hoos T. Advocating for environmental changes to increase access to parks: engaging promotoras and youth leaders. Health Promot Pract. (2013) 14:759–66. 10.1177/1524839912473303 23362333

[22] Bush DE Wilmsen C Sasaki T Barton-Antonio D Steege AL Chang C. Evaluation of a pilot promotora program for Latino forest workers in southern Oregon. Am J Ind Med. (2014) 57:788–99. 10.1002/ajim.22347 24890853

[23] Cramer ME Mollard EK Ford AL Kupzyk KA Wilson FA. The feasibility and promise of mobile technology with community health worker reinforcement to reduce rural preterm birth. Public Health Nurs. (2018) 35:508–16. 10.1111/phn.12543 30216526

[24] Furman L Matthews L Davis V Killpack S O'Riordan MA Breast for success: a Community–Academic collaboration to increase breastfeeding among high-risk mothers in Cleveland. Prog Community Health Partnersh. (2016) 10:341–53. 10.1353/cpr.2016.0041 28230542

[25] Harvey I Schulz A Israel B Sand S Myrie D Lockett M The Healthy Connections project: a community-based participatory research project involving women at risk for diabetes and hypertension. Prog Community Health Partnersh. (2009) 3:287–300. 10.1353/cpr.0.0088 20097990

[26] Ingram M Piper R Kunz S Navarro C Sander A Gastelum S. Salud Sí: A case study for the use of participatory evaluation in creating effective and sustainable community-based health promotion. Fam Community Health. (2012) 35:130–8. 10.1097/FCH.0b013e31824650ed 22367260

[27] Ingram M Schachter KA Sabo SJ Reinschmidt KM Gomez S De Zapien JG. A community health worker intervention to address the social determinants of health through policy change. J Prim Prev. (2014) 35:119–23. 10.1007/s10935-013-0335-y 24363179 PMC6742602

[28] Islam NS Wyatt LC Patel SD Shapiro E Tandon SD Mukherji BR. Evaluation of a community health worker pilot intervention to improve diabetes management in Bangladeshi immigrants with type 2 diabetes in New York City. Diabetes Educ. (2013) 39:478–93. 10.1177/0145721713491438 23749774 PMC3912744

[29] Islam NS Zanowiak JM Wyatt LC Chun K Lee L Kwon SC. A randomized-controlled, pilot intervention on diabetes prevention and healthy lifestyles in the New York City Korean community. J Community Health. (2013) 38:1030–41. 10.1007/s10900-013-9711-z 23813322 PMC3964609

[30] Islam NS Zanowiak JM Wyatt LC Kavathe R Singh H Kwon SC. Diabetes prevention in the New York City Sikh Asian Indian community: a pilot study. Int J Environ Res Public Health. (2014) 11:5462–86. 10.3390/ijerph110505462 24852392 PMC4053907

[31] Kutcher R Moore-Monroy M Bello E Doyle S Ibarra J Kunz S. Promotores as advocates for community improvement: experiences of the western states REACH Su Comunidad consortium. J Ambul Care Manage. (2015) 38:321–32. 10.1097/JAC.0000000000000073 26353024

[32] Marín A Carrillo L Arcury TA Grzywacz JG Coates ML Quandt SA. Ethnographic evaluation of a lay health promoter program to reduce occupational injuries among Latino poultry processing workers. Public Health Reports. (2009) 124(Suppl. 1):36–43. 10.1177/00333549091244S105 19618805 PMC2708655

[33] Marrone N Ingram M Somoza M Jacob DS Sanchez A Adamovich S. Interventional audiology to address hearing health care disparities: Oyendo Bien pilot study. Semin Hear. (2017) 38:198–211. 10.1055/s-0037-1601575 28522894 PMC5435479

[34] Michael YL Farquhar SA Wiggins N Green MK. Findings from a community-based participatory prevention research intervention designed to increase social capital in Latino and African American communities. J Immigr Minor Health. (2008) 10:281–9. 10.1007/s10903-007-9078-2 17665307

[35] Minkler M Garcia AP Williams J LoPresti T Lilly J. Sí se puede: using participatory research to promote environmental justice in a Latino community in San Diego, California. J Urban Health. (2010) 87:796–812. 10.1007/s11524-010-9490-0 20683782 PMC2937121

[36] Moore AA Karno MP Ray L Ramirez K Barenstein V Portillo MJ. Development and preliminary testing of a promotora-delivered, Spanish language, counseling intervention for heavy drinking among male, Latino day laborers. J Subst Abuse Treat. (2016) 62:96–101. 10.1016/j.jsat.2015.11.003 26738641 PMC4744478

[37] Nicolaidis C Mejia A Perez M Alvarado A Celaya-Alston R Quintero Y. Proyecto Interconexiones: pilot-test of a community-based depression care program for Latina violence survivors. Prog Community Health Partnersh. (2013) 7:395–401. 10.1353/cpr.2013.0051 24375180 PMC4845956

[38] Rios-Ellis B Nguyen-Rodriguez ST Espinoza L Galvez G Garcia-Vega M. Engaging community with promotores de salud to support infant nutrition and breastfeeding among Latinas residing in Los Angeles County: Salud con Hyland's. Health Care Women Int. (2015) 36:711–29. 10.1080/07399332.2014.900060 24625100

[39] Rios-Ellis B Espinoza L Bird M Garcia M D'Anna LH Bellamy L. Increasing HIV-related knowledge, communication, and testing intentions among Latinos: Protege tu Familia: Hazte la Prueba. J Health Care Poor Underserved. (2010) 21:148–68. 10.1353/hpu.0.0360 20675952

[40] Schwartz R Powell L Keifer M. Family-based risk reduction of obesity and metabolic syndrome: an overview and outcomes of the Idaho partnership for Hispanic health. J Health Care Poor Underserved. (2013) 24:129–44. 10.1353/hpu.2013.0106 23727970

[41] Simonsen SE Ralls B Guymon A Garrett T Eisenman P Villalta J. Addressing health disparities from within the community: community-based participatory research and community health worker policy initiatives using a gender-based approach. Women's Health Issues. (2017) 27(Suppl. 1):S46–53. 10.1016/j.whi.2017.09.006 29050658

[42] Suarez N Mendoza I Garrett S Ellerbeck EF Success of “Promotores de Salud” in identifying immigrant Latino smokers and developing quit plans. Int Public Health J. (2012) 4:343–53.

[43] Quesada J Hart LK Bourgois P Structural vulnerability and health: Latino migrant laborers in the United States. Med Anthropol. (2011) 30:339–62. 10.1080/01459740.2011.576725 21777121 PMC3146033

[44] Wallerstein N Duran B. Community-based participatory research contributions to intervention research: the intersection of science and practice to improve health equity. Am J Public Health. (2010) 100(Suppl. 1):S40–6. 10.2105/AJPH.2009.184036 20147663 PMC2837458

